# Multi-Stage Open Peer Review: Scientific Evaluation Integrating the Strengths of Traditional Peer Review with the Virtues of Transparency and Self-Regulation

**DOI:** 10.3389/fncom.2012.00033

**Published:** 2012-07-05

**Authors:** Ulrich Pöschl

**Affiliations:** ^1^Max Planck Institute for ChemistryMainz, Germany

**Keywords:** open evaluation, public peer review, open access publishing, interactive discussion, open peer commentary, transparency, self-regulation

## Abstract

The traditional forms of scientific publishing and peer review do not live up to all demands of efficient communication and quality assurance in today’s highly diverse and rapidly evolving world of science. They need to be advanced and complemented by interactive and transparent forms of review, publication, and discussion that are open to the scientific community and to the public. The advantages of open access, public peer review, and interactive discussion can be efficiently and flexibly combined with the strengths of traditional scientific peer review. Since 2001 the benefits and viability of this approach are clearly demonstrated by the highly successful interactive open access journal Atmospheric Chemistry and Physics (ACP, www.atmos-chem-phys.net) and a growing number of sister journals launched and operated by the European Geosciences Union (EGU, www.egu.eu) and the open access publisher Copernicus (www.copernicus.org). The interactive open access journals are practicing an integrative multi-stage process of publication and peer review combined with interactive public discussion, which effectively resolves the dilemma between rapid scientific exchange and thorough quality assurance. Key features and achievements of this approach are: top quality and impact, efficient self-regulation and low rejection rates, high attractivity and rapid growth, low costs, and financial sustainability. In fact, ACP and the EGU interactive open access sister journals are by most if not all standards more successful than comparable scientific journals with traditional or alternative forms of peer review (editorial statistics, publication statistics, citation statistics, economic costs, and sustainability). The high efficiency and predictive validity of multi-stage open peer review have been confirmed in a series of dedicated studies by evaluation experts from the social sciences, and the same or similar concepts have recently also been adopted in other disciplines, including the life sciences and economics. Multi-stage open peer review can be flexibly adjusted to the needs and peculiarities of different scientific communities. Due to the flexibility and compatibility with traditional structures of scientific publishing and peer review, the multi-stage open peer review concept enables efficient evolution in scientific communication and quality assurance. It has the potential for swift replacement of hidden peer review as the standard of scientific quality assurance, and it provides a basis for open evaluation in science.

## Introduction

The traditional ways of scientific publishing and peer review do not live up to the needs of efficient communication and quality assurance in today’s highly diverse and rapidly developing world of science. Besides high profile cases of scientific fraud, science, and society are facing a flood of carelessly prepared scientific papers that are locked away behind subscription barriers, dilute rather than enhance scientific knowledge, lead to a waste of resources and impede scientific and societal progress. On the other hand, the spread of innovative ideas and concepts is often delayed by inertia and obstruction in the hidden review process of traditional mainstream scientific journals (Pöschl, 2004).

Open access to scientific research publications is desirable for many educational, economic, and scientific reasons (Max Planck Society, 2003; David and Uhlir, 2005; European Commission and German Commission for UNESCO, 2008), and it provides major opportunities for the improvement of scientific communication, quality assurance, and evaluation (Bodenschatz and Pöschl, 2008; Pöschl and Koop, 2008; Pöschl, 2010b):

(1) Open access is fully compatible with traditional peer review, and in addition it enables interactive and transparent forms of review and discussion open to all interested members of the scientific community and the public (open peer review).
(2) Open access gives reviewers more information to work with, i.e., it provides unlimited access to relevant publications across different scientific disciplines and communities (interdisciplinary scientific discussion and quality assurance).
(3) Open access facilitates the development and implementation of new metrics for the impact and quality of scientific publications (combination of citation, download/usage, commenting, and ranking by various groups of readers and users, respectively; Bollen et al., 2009).
(4) Open access helps to overcome the obsolete monopoly/oligopoly structures of scientific publishing and statistical analysis of publication contents and citations/references, which are limiting the opportunities for innovation in scientific publishing and evaluation.

As demonstrated below, the effects and advantages of open access, public review, and interactive discussion can be efficiently and flexibly combined with the strengths of traditional scientific publishing and peer review (Pöschl, 2009a, 2010a,b). Unlike other, more radical proposals of how to change and improve scientific quality assurance, the interactive open access publishing approach introduced by the international scientific journal Atmospheric Chemistry and Physics (ACP) conserves the strengths of traditional peer review while overcoming its major weaknesses. This approach is compatible with the structures of traditional scientific publishing and quality assurance, and thus it enables an efficient transition from the operational but sub-optimal past of subscription-based journals and hidden peer review to the future of free exchange and transparent evaluation of scientific information on the internet.

## Multi-Stage Open Peer Review

**Figure 1:**
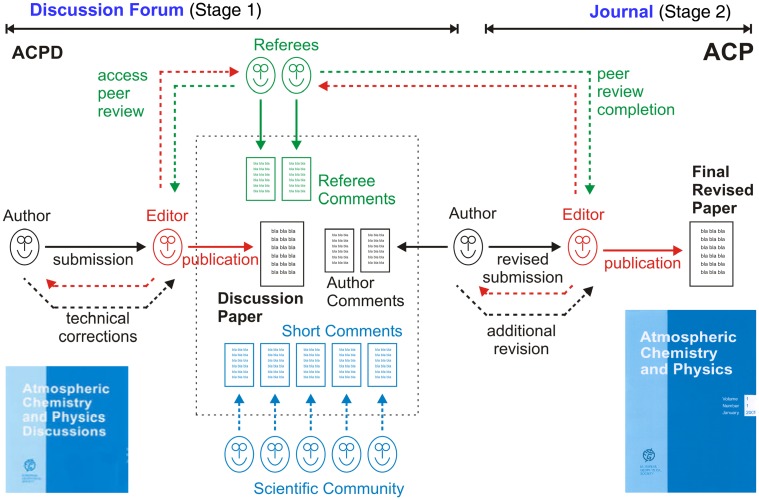
**Multi-stage open peer review as practiced in the scientific journal Atmospheric Chemistry and Physics (ACP) and its discussion forum Atmospheric Chemistry and Physics Discussions (ACPD)**. Solid and dashed arrows indicate required and optional processes and interactions between author, editor, referees, and scientific community.

So far, the arguably most successful alternative to the closed peer review of traditional scientific journals is the multi-stage open peer review practiced by ACP and a growing number of interactive open access sister journals of the European Geosciences Union (EGU) and Copernicus Publications (Pöschl, 2010b). As detailed below (see Atmospheric Chemistry and Physics and the European Geosciences Union), ACP is by most if not all standards more successful than comparable scientific journals with traditional or alternative forms of peer review (editorial statistics, publication statistics, citation statistics, economic costs, and sustainability). The multi-stage open peer review of ACP is based on a two-stage process of open access publishing combined with multiple steps of peer review and interactive public discussion as illustrated in Figure 1.

In the first stage, manuscripts that pass a rapid pre-screening (access review) are immediately published as “discussion papers” in the journal’s discussion forum (Atmospheric Chemistry and Physics Discussions, ACPD). They are then subject to interactive public discussion for a period of 8 weeks, during which the comments of designated referees, additional comments by other interested members of the scientific community, and the authors’ replies are also published alongside the discussion paper. While referees can choose to sign their comments or remain anonymous, comments by other scientists (registered readers) are automatically signed. In the second stage, manuscript revision and peer review are completed in the same way as in traditional journals (with further rounds of review and revision where required) and, if accepted, final papers are published in the main journal. To provide a lasting record of review and to secure the authors’ publication precedence, every discussion paper, and interactive comment remains permanently archived and individually citable.

The multi-stage peer review and publication process of ACP effectively resolves the dilemma between rapid scientific exchange and thorough quality assurance, and it offers a win-win situation for all involved parties (authors, referees, editors, publishers, readers/scientific community). The primary positive effects and advantages compared to the traditional forms of publication with closed peer review are:

1. The discussion papers offer free speech and rapid dissemination of novel results and original opinions, without revisions that might delay or dilute innovation (authors’ and readers’ advantage).
2. The interactive peer review and public discussion offer direct feedback and public recognition for high quality papers (authors’ advantage); they prevent or minimize the opportunity for hidden obstruction and plagiarism (authors’ advantage); they provide complete and citable documentation of critical comments, controversial arguments, scientific flaws, and complementary information (referees’ and readers’ advantage); they reveal deficiencies and deter submissions of carelessly prepared manuscripts, thus helping to avoid/minimize the waste of time and effort for deficient submissions (referees’, editors’, publishers’, and readers’ advantage).
3. The final revised papers offer a maximum of scientific information density and quality assurance achieved by full peer review (with optional anonymity of referees) and revisions based on the referees’ comments plus additional comments from other interested scientists (readers’ advantage).

Readers who are primarily interested in the quintessence of manuscripts that have been fully peer reviewed and approved by referees and editors can simply focus on the final revised paper (or, indeed, its abstract) published in the journal and neglect the preceding discussion papers and interactive comments published in the discussion forum. Thus the two-stage publication process does not inflate the amount of time required to maintain an overview of final revised papers. On the other hand, readers who want to see original scientific manuscripts and messages before they are influenced by peer review and revision, and who want to follow the scientific discussion between authors, referees, and other interested scientists, can browse the papers and interactive comments in the discussion forum.

The possibility of comparing a final revised paper with the preceding discussion paper and following the interactive peer review and public discussion also facilitates the evaluation of individual publications for non-specialist readers and evaluators. The style and quality of interactive commenting and argumentation provide insights that go beyond, and complement, the information contained in the research article itself.

The multi-stage process of review and publication stimulates scientists to prove their competence via individual high quality papers and their discussion, rather than just by pushing as many papers as possible through journals with closed peer review and no direct public feedback and recognition for their work. Authors have a much stronger incentive to maximize the quality of their manuscripts prior to submission for peer review and publication, since experimental weaknesses, erroneous interpretations, and relevant but unreferenced earlier studies are more likely to be detected and pointed out in the course of interactive peer review and discussion open to the public and all colleagues with related research interests.

Moreover, the transparent review process prevents authors from abusing the peer review process by delegating some of their own tasks and responsibilities to the referees during review and revision behind the scenes. Referees often make substantial contributions to the quality of scientific papers, but in traditional closed peer review their input rarely receives public recognition. The full credit for the quality of a paper published in a traditional journal generally goes to the authors, even when they have submitted a carelessly prepared manuscript that has taken a lot of time and effort on the part of the referees, editors, and publishers to turn it into a good one. While peer review depends crucially on the availability and performance of referees, it has traditionally offered little reward for those providing careful and constructive reviews. In public review, however, referees’ arguments are publicly heard and, if comments are openly signed, referees can also claim authorship for their contribution.

Note that most of the effects and advantages outlined above are not fully captured by alternative approaches where interactive commenting and public discussion occur only after formal peer review and final publication of scientific papers or where the discussion paper and interactive comments are removed after publication of the final revised paper (see Key features of multi-stage open peer review as practiced by ACP).

Overall, the interactive open access publishing philosophy emphasizes the value of free speech and efficient public exchange and scrutiny of scientific results in line with the principles of critical rationalism and open societies. Accordingly, editors and referees are supposed to critically comment and evaluate manuscripts, to help authors improve their manuscripts, and to eliminate clearly deficient manuscripts. However, authors shall not be forced to adopt the editors’ or referees’ views and preferences. Instead, the readers shall be able to make up their own mind in view of the public review and discussion. In case of doubt, editorial decisions shall favor free speech of scientists, and in the end, scientific progress; history shall tell if – or to which degree – they were right. In scientific research, the line between fundamental flaws and major innovations can be fine, and the multi-stage process of interactive open access publishing and peer review enables efficient balancing and differentiation between potentially misleading hypotheses and innovative theories even in highly controversial cases (Pöschl, 2004, 2010b).

## Atmospheric Chemistry and Physics and the European Geosciences Union

The interactive open access journal Atmospheric Chemistry and Physics (ACP^1^), founded in 2001, demonstrates that multi-stage open peer review enables much more efficient quality assurance than traditional closed peer review. ACP is run by the European Geosciences Union (EGU^2^), the open access publisher Copernicus^3^, and a globally distributed network of scientists (∼130 co-editors coordinated by an executive committee of five). Manuscripts are normally handled by an editor who is familiar with the specific subject area of the submitted work and independently guides the review process. Details about the largely automated handling and editor assignment of submitted manuscripts are given below (see Key features of multi-stage open peer review as practiced by ACP) and on the journal website. The origin and development of interactive open access publishing as practiced by ACP and EGU/Copernicus are specified in a recent anniversary publication (Pöschl, 2010c, 2011; Copernicus, 2011)^4^.

Currently ACP publishes about 800 papers per year (∼13,000 double column print pages), which is similar to the volume of traditional major journals in the fields of chemistry and physics (ISI Science Citation Index, Journal Citation Report, 2010). On average, each paper receives four interactive comments, and about one in five papers receives a comment from the scientific community in addition to the comments from designated referees. In total, there are typically 0.5 pages of interactive comments per page of original discussion paper, i.e., the volume of interactive comments amount to as much as ∼50% of the volume of discussion papers. The interactive comments show the full spectrum of opinions in the scientific community, ranging from harsh criticism to open applause (sometimes for the same discussion paper), and they provide a wealth of additional information and evaluation that is available to everyone.

About three out of four referee comments are posted without the referee’s name, showing that most referees in the scientific community of ACP prefer anonymity. There are, however, interesting differences between sub-disciplines: on average about 20% of theoreticians and computer modelers sign their referee comments, while only 10% of the laboratory and field experimentalists do so. It appears that modelers more often provide suggestions and ideas for which they like to claim authorship as a reward. The anonymous referee comments are generally also very constructive and substantial. The ACP editors do not actively moderate the public discussions but reserve the right to delete abusive or inappropriately worded comments. Out of the nearly 20,000 interactive comments that have been posted so far, only a handful were removed or replaced because of inappropriate wording, which demonstrates efficient self-regulation by transparency.

Some colleagues have expressed concerns that referees may lose their independence by having access to the comments from fellow referees and from the public. Indeed, referees with limited capacities occasionally seem to duplicate or refer to earlier comments without making up their own mind, but this is fairly easy to recognize and to take into account by editors and readers. Much more often, however, referees constructively build on or contradict earlier comments, which enhances the efficiency of review and discussion substantially. In theory, the independence of referees could be maintained by keeping submitted referee comments non-public until all referees have submitted their comments and these are all together published at the same time. In practice, however, this would cause unnecessary delays (“waiting for the last referee”) and stifle rather than promote interactive discussion. Overall, experience shows that the advantages of enabling direct interaction between referees clearly outweigh the disadvantages.

The average rate of public commenting in addition to the designated referees’ and authors’ comments specified above (∼20%) may appear low at first sight. It is, however, by an order of magnitude (factor ∼10) higher than in journals with post-peer review online commenting and in traditional journals without online commenting (about 1–2%; Müller, 2008; Pöschl and Koop, 2008; Pöschl, 2010b). Discussion papers reporting controversial findings or innovations attract many interactive comments (up to 30 and more, see “Most commented papers” in the ACPD online library^5^. As expected, non-controversial papers usually elicit comments only from the designated referees. Why would scientists invest effort and time commenting on papers which they find interesting but not controversial?

In most scientific disciplines and journals (certainly in the fields of physics, chemistry, and biology with which the author is well acquainted) it is notoriously difficult to assign a couple of competent referees to every manuscript submitted for publication. In fact, this is the main bottleneck of peer review and scientific quality assurance, and most journal editors have to apply lots of manpower and electronic tools (invitation and reminder emails, etc.) to obtain a couple of referee comments per manuscript. Accordingly, the initiators and editors of ACP are quite satisfied with the overall number and volume of interactive comments. Higher rates of commenting were not expected and are not required to stimulate self-regulation mechanisms of scientific quality assurance (Pöschl, 2004, 2010a,b).

The editorial and citation statistics of ACP clearly demonstrate that multi-stage open peer review indeed facilitates and enhances scientific communication and quality assurance. The journal has relatively low rejection rates (∼15% as opposed to ∼50% in comparable traditional journals, Schultz, 2010), but only a few years after its launch ACP had already achieved top reputation and visibility in the scientific community. Accordingly, it quickly reached and maintained one of the highest ISI impact factors of several 100 journals indexed across the disciplines of atmospheric sciences, geosciences, and environmental sciences (JIF ≈ 5). These figures clearly confirm that anticipation of public peer review and discussion deters authors from submitting low-quality manuscripts and, thus, relieves editors and referees from spending too much time on deficient submissions. This is particularly important, because refereeing capacities are the most limited resource in scientific publishing and quality assurance. The high efficiency, robustness, and predictive validity of the multi-stage open peer review process of ACP have been confirmed in a series of dedicated studies by evaluation experts from the social sciences (Bornmann and Daniel, 2010a,b; Bornmann et al., 2010, 2011a,b).

Since its launch in 2001, the number of articles published in ACP has increased rapidly. The high and increasing rates of submission, publication, and citation show that the scientific community values the open access, high quality, and interactive discussions of ACP. They confirm that there is a demand for improved scientific publishing and quality assurance, and that the interactive open access journal concept of ACP meets this demand. Today ACP is the largest journal in the field of atmospheric sciences and one of the largest across the fields of environmental and geosciences, offering at the same time top visibility and low rejection rates (2/5 year impact factors 5.4/5.8, rejection rate 15%, 12,000 pages in 2010). The combination of top visibility with high volume and low rejection rate, i.e., high efficiency by self-regulation, is a fairly unique achievement in the world of scientific publishing, where the most visible journals traditionally had relatively small volumes and high rejection rates (Copernicus, 2011; Pöschl, 2011).

Following up on the successful development of ACP, the EGU, and Copernicus have launched and are operating over a dozen of interactive open access sister journals in the geosciences and related disciplines, and more are in the pipeline^6^:

– Atmospheric Chemistry and Physics (ACP)^7^,
– Atmospheric Measurement Techniques (AMT)^8^,
– Biogeosciences (BG)^9^,
– Climate of the Past (CP)^10^,
– Drinking Water Engineering and Science (DWES)^11^,
– Earth System Dynamics (ESD)^12^,
– Earth System Science Data (ESSD)^13^,
– Geoscientific Instrumentation, Methods and Data Systems (GI, geoscientific-instrumentation-methods-and-data-systems.net),
– Geoscientific Model Development (GMD)^14^,
– Hydrology and Earth System Sciences (HESS)^15^,
– Ocean Science (OS)^16^,
– Social Geography (SG)^17^,
– Solid Earth (SE)^18^,
– The Cryosphere (TC)^19^.

**Figure 2:**
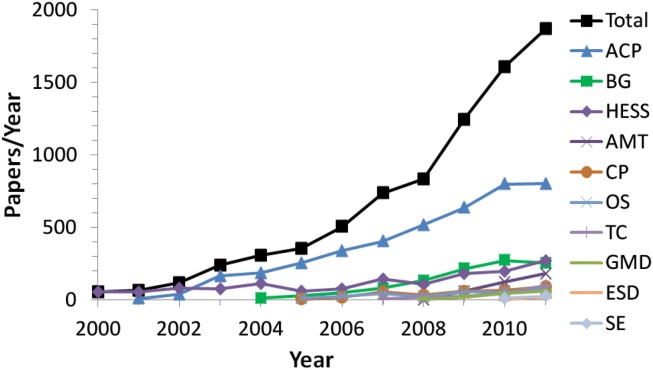
**Number of papers published per year in the interactive open access journals of the European Geosciences Union (EGU)**.

Figure 2 illustrates the growth of ACP and the other EGU interactive open access journals over the past decade^20^. The wide range of different topics and scientific communities covered by the EGU interactive open access journals demonstrates that multi-stage open peer review is suitable for any kind of topical scientific journal. For example, the community of cryospheric sciences is much smaller than that of atmospheric sciences, but the development of the cryospheric science journal (TC) proceeds at least as well as that of the atmospheric science journals (ACP and AMT). The first journal impact factor of TC was already the highest in its field. The journal Hydrology and Earth System Sciences (HESS) had already existed as a subscription-based journal with traditional peer review before it was converted into an interactive open access journal. Soon after the transition, the journal experienced a substantial increase of submissions, publications, and citations, demonstrating that traditional journals can be successfully converted into interactive open access journals. Three other open access journals published by EGU (Annales Geophysicae, Natural Hazards, and Earth System Sciences, Non-linear Processes in Geophysics) have maintained traditional peer review up to now. In view of the more successful development of the interactive open access journals, however, they are planning to introduce multi-stage open peer review as well.

The multi-stage open peer review concept of ACP has also been adopted by the e-journal Economics^21^ which was launched in 2007 and involves some of the most prominent institutions and scientists in the field of economics. Alternative concepts of public peer review and interactive discussion are pursued by the open access publications Journal of Advances in Earth System Modeling (JAMES; since 2008)^22^, PLoS One^23^, Biology Direct^24^, Electronic Transactions of Artificial Intelligence (ETAI; since 1997)^25^, and Journal of Interactive Media in Education (JIME; since 1996)^26^. Differences between the peer review concepts of these publications and ACP will be addressed and discussed below (see Key features of multi-stage open peer review as practiced by ACP and Comparison to Earlier Initiatives with Two- or Multi-Stage Open Peer Review). In short, approaches where interactive commenting and public discussion are not fully integrated with formal peer review by designated referees tend to be less successful.

## Financing and Sustainability of Interactive Open Access Publishing

Atmospheric Chemistry and Physics and its EGU/Copernicus sister journals prove not only the scientific but also the economic viability and sustainability of interactive open access publishing and peer review. The journals were launched and are operated by the independent scientific society EGU and by the small commercial enterprise Copernicus without public subsidies, private donations, or venture capital as involved in the start-up and operation of other successful open access publishers like PLoS and BioMed Central. After several years of operation, ACP and its sister journals have recovered the financial investments of EGU and Copernicus during the start-up phase, and they now deliver a surplus which supports the start-up of new journals by the scientific society as well as a healthy growth of the commercial publisher generating dozens of new jobs.

By developing and applying efficient software tools for the handling of manuscripts (submission, peer review and commenting, typesetting/production, and distribution), and because minimal time and effort is wasted on carelessly prepared papers (high quality of submissions and low rejection rates as detailed above), Copernicus is able to produce top quality publications at comparatively low cost. The publication service charges are of the order of one hundred Euros per page in final double column format, i.e., about one thousand Euros for an average paper with a length of about ten pages. The service charges cover the review support from the editorial office, free use of color figures and online supplementary materials (data, pictures, movies etc.), typesetting of both the discussion and the final version of the paper, archiving and distribution of papers, and interactive comments (maintenance of websites and servers, electronic copies for open archives, paper copies for copyright libraries, etc.) and overheads. In agreement between the publisher (Copernicus) and the scientific society (EGU council and publications committee), the service charges are adjusted to cover the full costs of publishing, including all the tasks and services outlined above, and to generate a modest surplus for the scientific union: ∼10% of the annual financial turnover (currently about three million Euros). The surplus is re-invested in publication development (new journals and services) and it helps to run the membership and outreach activities of EGU, which is a non-profit organization. Like the other scientific officers of the union, editors do their work unpaid on a purely voluntary basis. Following up on the questions and suggestions of a reviewer of this manuscript, I would like to clarify that neither I nor any other editor of ACP and the other EGU interactive open access journals have had any income from the journals that we edit as a voluntary community service. In fact, we pay regular registration fees of up to 500 EUR to attend the annual general assembly and scientific conference of our union (EGU), where the editorial board meetings take place. The separation of financial and scientific interests seems important in the context of peer review, and the ACP/EGU experience demonstrates that a purely voluntary approach on the scientific editors’ side is sustainable and compatible with efficient operation of open access journals by a commercial publisher.

For each paper published in ACP, the service charges are levied from the authors or paid by their scientific institution. Since 2008 the German Max Planck Society (MPG)^27^ and the French Centre National de Recherche Scientifique (CNRS)^28^ have contracts with Copernicus for automated coverage of service charges incurred by their scientists. Other scientific institutions are likely to follow these examples, and many national and international research organizations and funding agencies pursue complementary ways of covering open access service charges for their scientists and projects. Like other open access publishers, Copernicus, and EGU are ready to cover the costs for up to 10% of the papers published each year, if the authors are unable to pay the service charges (e.g., authors without institutional support or institutions from less developed countries). Currently, most papers published in ACP originate from Europe (∼50%) and North America (∼30%), but the proportion of papers originating from Asia and other regions is increasing.

The ACP open access publication service charges compare quite favorably with the charges levied by other comparable scientific journals and publications:

1. Other major open access publishers such as BioMed Central and the Public Library of Science (PLoS) typically charge more than 1,000 EUR for traditional single-stage journal publications.
2. Traditional publishing groups like Springer charge 2,000 EUR for making individual publications in traditional subscription journals freely available online (“open choice”), i.e., they levy 2,000 EUR per online open access paper in addition to charging libraries and other subscribers for access to the journal in which it appears.
3. In the traditional scientific publishing business, where some journals do not only limit access to subscribers or sell articles on a pay-per-view basis but also request additional publication charges from authors (up to several hundred US dollars per page or color figure), the total turnover, and public costs amount to several thousand US dollars per paper. The annual turnover of journal publishing in the sector of science, technology, and medicine (STM) amounts to around seven billion USD per year, and some of the traditional publishers – led by Elsevier with a market share of about 30% – make operating profits of up to 30% and more. Note that a large proportion of the turnover and profit in STM publishing comes from packaging and selling publicly funded research results that are peer reviewed by publicly funded scientists to publicly funded institutions of education and research (Economist Academic Publishing, 2011; Golden and Schultz, 2012).

In view of these facts, ACP authors and the ACP scientific community have had little difficulty in accepting and paying average service charges of about one thousand Euros per paper to make ACP and its sister journals sustainable. Overall, ACP and its interactive open access sister journals prove that top quality (interactive) open access publishing and peer review can be realized and sustained by scientific societies and (small) commercial publishers with tightly limited budgets and without public subsidies, private donations or venture capital. Indeed, ACP, EGU, and Copernicus demonstrate how STM publishing at large can and will hopefully soon manage a swift transition from the past of print-based subscription barriers into the future of an internet-based open access environment.

## Key Features of Multi-Stage Open Peer Review as Practiced by ACP

The following key features of the ACP multi-stage open peer review system help ensure maximum efficiency of scientific exchange and quality assurance, making it more successful than most other forms of closed or open peer review:

1. Publication of discussion papers before full peer review and revision: free speech, rapid publication, and public accountability of authors for their original manuscript foster innovation and deter careless submissions.
2. Integration of public peer review and interactive discussion prior to final publication: attract more comments than post-peer review commenting, enhance efficiency, and transparency of quality assurance, maximize information density of final papers.
3. Optional anonymity for designated referees: enables critical comments and questions by referees who might be reluctant to risk appearing ignorant or disrespectful – especially when providing a voluntary community service in which they have little to gain for investing lots of effort and time.
4. Archiving, public accessibility, and citability of every discussion paper and interactive comment: ensure documentation of controversial scientific innovations or flaws, public recognition of commentators’ contributions, and deterrence of careless submissions.

Combining all of the above features and effects is the basis for the great success of ACP and its sister journals. Missing out on one or more of these features is the main reason why most if not all alternative forms of peer review practised in other initiatives for improving scientific communication and quality assurance have been less successful (less commenting, lower impact/visibility, higher rejection rates, larger waste of refereeing capacities, etc.).

For example, the release of a “pre-publication history” and/or the opportunity for “peer commentary” after completion of the actual peer review and publication of the final revised manuscript as practiced by the BMC medical journals of BioMed Central^29^ as well as the journals Behavioral and Brain Sciences^30^ and Psychology^31^ are very useful advances and improvements compared to traditional journal publishing, but they miss some of the above features and advantages. Controversial scientific innovations or flaws in papers rejected after peer review are not documented for the public and scientific community. Moreover, the completion of peer review and revision before publication and public discussion of a manuscript does not allow interested members of the scientific community to have any input to the revision and the final editorial decision. Obviously, “post-commenting” after peer review is much less attractive to scientists than commenting in the course of peer review. The latter allows individual scientists to support and influence the conclusions and publications of their colleagues, e.g., by pointing out related earlier findings and studies which the authors can still include in the reference list of the manuscript thus in standard citation analyses. In contrast, post-commenting after final publication does neither enable the commentator to influence the final publication, nor does it allow the authors to improve their publication along the lines suggested by the commentators. Accordingly, potential commentators have not only less incentive to invest effort and time in contributing to their colleagues’ and competitors’ work; they also have to worry that critical comments might just be regarded as a devaluing critique rather than a helpful contribution. This fairly straightforward consideration is supported by the fact that most journals with post-commenting receive fewer comments from the scientific community (Müller, 2008). For example, only one of ∼20 papers published in PLoS One receives a comment from the scientific community (as opposed to one of ∼5 in ACP), although PLoS offers more advanced and easier to use commenting tools and tries to advertise and promote the commenting more actively than ACP.

For several reasons also the “open peer review trial” of the Nature magazine in 2006 was not a good example and measure for the engagement of scientists in interactive commenting and public peer review on the internet. In that experiment, neither the authors of an article nor their colleagues and readers had much of an incentive to participate in the public discussion. The authors had to accept that their article was exposed in parallel to public scrutiny as well as to a closed peer review process where the referee comments remain non-public and where most of submitted manuscripts are rejected not because of a lack of scientific quality but because they are not deemed sufficiently exciting for the interdisciplinary audience of the magazine (ca. 93% rejection rate)^32^. For the likely outcome that a manuscript would not pass the closed peer review, it was not clear whether and in which form the rejected manuscript and the public comments would remain publicly accessible. As one might have imagined beforehand, this is not a very attractive perspective for scientists trying to get recognition for their most exciting results. Similarly, colleagues and readers had little incentive to formulate and post substantial comments, because their contributions would just have been an addendum to the closed peer review proceeding in parallel and would likely disappear afterward. Fortunately, the publishers of Nature seem to have realized that permanent archiving and citability are key features of scientific exchange, and they have launched a more promising initiative titled Nature Precedings. There manuscripts can be published, openly discussed and archived in a similar way as in the discussion forums of interactive open access journals^33^.

Unfortunately, however, it seems that the paramount importance of archiving and citability of manuscripts and comments has not yet been fully recognized by scientific publishers and societies. Following up on the success and leadership of the EGU in interactive open access publishing and peer review, the American Geophysical Union (AGU) has recently also engaged in experiments with “open peer review.” Instead of building on the very positive experience and success of the European sister society, however, AGU seems to follow the tracks of the unsuccessful earlier trial of Nature. Specifically, AGU announced that the discussion paper and all interactive comments shall be deleted after completion of the peer review process and final acceptance or rejection of the revised manuscript (Albarede, 2009). This line was also followed in the JAMES, which had originally adopted the interactive open access journal concept of ACP but then abandoned the archiving of discussion papers in their discussion forum (JAMES-D) and was recently taken over by AGU. If AGU were to continue the approach of erasing discussion papers and comments, they would largely miss out on the effects detailed under point 4 above, and it appears questionable that the perspective of deletion after a couple of months will attract substantial commenting from the scientific community. Hopefully, the proponents of the AGU experiment will realize that the deletion of scientific comments is not only a discouragement for potential commentators but also a regrettable underestimation of the value of scientific discussion and discourse in the history and progress of science.

As outlined on the web pages of ACP/EGU, the permanent archiving of discussion papers can occasionally lead to inconveniences for authors and other parties involved in the review and publication process. Overall, however, the advantages of permanent archiving clearly outweigh the potential disadvantages^34^^,^^35^.

For the following reasons it would be neither appropriate nor possible to delete discussion papers after they have been published online:

(1) The deletion of published materials is incompatible with the virtues of traceability and reliability that are central to science and scientific publishing in general, and to the interactive open access publishing approach of ACP/EGU in particular. Deleting published scientific information is against the very nature of science.
(2) The deletion of discussion papers and comments would discourage potential commentators, and it would imply a disregard for the value of scientific discourse (Pöschl, 2010b, pp. 305–306).
(3) The use of digital object identifiers (DOI) entails legal obligations of ensure permanent archiving and accessibility.
(4) Even if it were desirable, it would be practically impossible to “unpublish” a discussion paper published in ACPD. Upon online publication, the papers are copied into multiple electronic repositories. Moreover, referees, readers, and other internet users can and do download copies for storage at arbitrary locations that are beyond the control of any the publisher. Therefore, a published paper can be formally withdrawn/retracted by publication of a commentary analogous to stating the reasons like in traditional print journals. It can, however, not be “unpublished” by deletion from the web pages and archives of the journal.

One of the central aims of interactive open access publishing is high efficiency in scientific communication and quality assurance. As detailed in the attached articles, the average quality and visibility of ACP, and its sister journals are higher than those of most comparable journals while the rejection rates are lower. The highly efficient mechanism of review, publication, and self-regulation would hardly work if authors could submit manuscripts at any rate and simply delete published discussion papers if the public peer review and editorial decision were not favorable (or for any other reason).

Experience and rational thinking suggest that multi-stage open peer review should be applicable and beneficial for journal publications in most if not all disciplines of scientific research (STM as well as social sciences, economics, and humanities). For consistency and traceability, discussion papers, and interactive comments should generally remain archived and citable as published, and they should be regarded as proceedings-type publications. Due to the proceedings character of discussion papers, the authors of revised manuscripts that may not have been accepted for final publication in the interactive open access journal to which they had originally been submitted can still pursue review and publication in alternative journals. As indicated above, such aspects are particularly important with regard to highlight magazines or journals in which the review process is not only aimed at ensuring scientific quality but also at high selectivity with regard to interdisciplinary relevance and visibility, which entails low probability of acceptance even for manuscripts of high quality (see Nature trial).

In addition to the above general features, the following specific procedural aspects have turned out to be important for the practical implementation and effectiveness of interactive open access publishing and peer review:

### Editor assignment

For the assignment of a newly submitted manuscript to a handling editor, the online editorial office automatically sends invitation letters to all editorial board members covering the relevant subject areas (based on index terms selected by authors). Depending on competence and availability, each editorial board member can then decide if s/he wants to take editorship (first come, first served; every board member is expected to handle at least six submissions per year). If no handling editor can be found via the automated assignment process, the authors and the executive editors are informed and asked to directly contact individual board members if they are ready to take editorship. This second line of editor assignment in ACP is similar to the regular editor assignment procedure in the open access journal Biology Direct^36^. There it is up to the authors to find and motivate an editorial board member to guide the review process for their paper, and the manuscript is effectively rejected if none of the board member agrees.

### Access review

Prior to publication in the discussion forum, the editor is asked to evaluate whether the submitted manuscript is within the scope of the journal and whether it meets basic quality criteria. If necessary, the editor may consult referees for a rapid and preliminary initial rating of the manuscript^37^. The editor or referees can request/suggest minor technical corrections and adjustment (typing errors, clarifications, etc.). Further requests for revision of the scientific contents are not allowed at this stage of the review process but shall be expressed in the interactive discussion following publication of the discussion paper. For rapid processing and in order to save refereeing capacities the editor shall normally perform the access review without the referees, unless their advice is urgently needed or the authors have requested their involvement. In a statement or cover letter accompanying the submitted manuscript, the authors can indicate if they have any preference on involving the referees already in the access review. Obviously, the involvement of referees can lead to delays, but on the other hand the authors may want to receive a preliminary rating and suggestions for minor corrections prior to publication of the discussion paper.

### Final response and review completion

In the final response phase at the end of the interactive public discussion, the authors shall respond to all comments. The editor has the opportunity of adding comments and suggestions, but normally editorial decisions and recommendations should not be taken and expressed before the authors have responded to all comments (“audiatur et altera pars”). Instead, it shall be up to the authors to decide if they want to pursue final publication and how they shall revise their manuscript in view of the public review and discussion (self-regulation once again). Depending on the situation, they can but need not ask and wait for the editor to give advice on how to proceed and if a revised version is likely to be accepted for final publication. After receiving critical feedback, mature, and responsible scientists should normally know best how to revise their manuscript. Indeed, the improvements upon revision of a manuscript after public discussion often go far beyond the requests and suggestions expressed by the referees. Premature interference by the editor would likely reduce rather than enhance the authors’ motivation for improving the manuscript upon revision. Moreover, premature editorial recommendations published by the editor before seeing the authors’ final response and the revised manuscript could potentially bias the final decision about acceptance or rejection.

After receiving the revised manuscript the editor has a complete picture, can check if all comments and suggestions have been properly taken into account, and can suggest or request further improvements. If required, the process of review and revision can be iterated with the help of referees. So far, such iterations of peer review as well as appeal procedures in case of controversial editorial decisions have not been handled in public to avoid unnecessary complications. In the end, however, the discussion forum can and shall be used to explain editorial decisions in a rational and transparent way as illustrated by the following example (Pöschl, 2009b)^38^:

Currently, the editorial guidelines of ACP encourage editors to publish scientifically useful referee-author exchanges from non-public part of peer review completion in similar ways as in the exemplary case cited above. In the future, intermediate manuscript versions and related comments from the access review or the review completion shall be automatically made available upon publication of a manuscript in ACPD or ACP, respectively (analogous to pre-publication history available in BMC medical journals). If, however, a newly submitted manuscript is not accepted for publication in ACPD or a revised manuscript is not accepted for publication in ACP, the manuscript, and related comments shall be kept confidential in order to avoid escalation of scientific disputes and to maintain the authors’ opportunity of pursuing publication in alternative publishing venues (European Geosciences Union, 2010).

## Comparison to Earlier Initiatives with Two- or Multi-Stage Open Peer Review

Following up on the requests of a referee in the peer review of this manuscript, the following paragraphs provide a detailed comparison to earlier initiatives with similar concepts and a discussion of potential reasons for different developments. During the initiation and planning of ACP and its interactive journal concept in the years 2000 and 2001, I was looking for – but was unable to find – similar initiatives to compare with and learn from (Pöschl, 2004). It was only at an e-publishing workshop of the Max Planck Center for Information Management in May 2002 that I learned of a similar initiative launched as early as 1996: the JIME^39^. Coming from a completely different scientific background, the founders of JIME had designed and realized a similar concept of multi-stage open peer review with public discussion. Unfortunately, however, JIME attracted only a small number of publications and seems not to have inspired the foundation of similar journals in related fields of science and humanities. Despite the overall conceptual similarities, JIME does not show some of the key features of the ACP interactive journal concept. In particular, the “private open peer review” of JIME foresees a non-public exchange of arguments between referees and authors, which is opened to the public only after approval by the editor. This seems to limit the publication and documentation of controversial scientific innovations or flaws much more than the “access peer review” of ACP (quick go/no-go decision essentially without non-public exchange of arguments between authors and referees). Moreover, all referees are named and no anonymous referee comments are allowed in JIME, which is likely to limit and inhibit critical review and discussion. These differences may appear subtle at first sight, but they are highly relevant for the practical operation of a scientific journal and may be decisive for its success and acceptance in the target scientific community.

After JIME, I got to know about another early online publication format with a two-stage open peer review process: the ETAI^40^ launched in 1997. Similar to JIME, ETAI attracted a series of special issues related to conferences or projects, but the number of individually submitted articles remained small. Regular operations stopped in 2002, but the ETAI home page indicates plans for a re-launch. As described by Sandewall (1997, 2006, 2012), the open peer review process of ETAI does not integrate but separate the two major aims of peer review, namely, to improve the quality of submitted manuscripts and to establish certain quality standards. The first stage is an interactive public discussion which invites questions, comments, and suggestions from the scientific community, but it does not involve designated referees, and all participants are openly named. In a second stage, anonymous referees decide about acceptance of the revised manuscript for ETAI, and further rounds of revision are normally not allowed. These features of ETAI bear similarities to the unsuccessful trial of open peer review by Nature magazine in 2006, and they are in stark contrast to the ACP review process, where the referees contribute to the interactive public discussion and have an option of staying anonymous, and the peer review process can be continued iteratively like in traditional journals. For the authors and readers of ETAI it seems not clear, if the openly named participants of the interactive public discussion in the first stage of the review process might also serve as an anonymous referees in the second stage. It seems rather unattractive for authors to post their manuscript for open discussion and scrutiny by the scientific community, and to have only one chance of revision before anonymous referees who may or may not have been involved in the preceding discussion are expected to make a “pass/fail decision” (Sandewall, 2012). In the relatively few review processes that have actually been completed in ETAI so far (several dozens in the time frame of 1997–2002), all involved parties seem to have requested exceptions, i.e., anonymity in the interactive public discussion and iterative revisions in the second stage of review (Sandewall, 2012). Both of these “exceptional” features are key elements of the successful ACP approach. From long-term experience with several thousand review processes completed in ACP since 2001, we know that these features are vital for the large success of the EGU interactive open access journals, and I would argue that they might be critical for the limited success of ETAI. In any case, the ACP/EGU approach of multi-stage open peer review is aimed at integrating rather than separating the processes of interactive public discussion and classical peer review as well as the aims of manuscript improvement and quality control.

The limited success of JIME and ETAI compared to ACP demonstrates the difficulties of practical implementation and the importance of the conceptual aspects and subtleties outlined above (see Key features of multi-stage open peer review as practiced by ACP). Nevertheless, the basic aims and principles of JIME, ETAI, and ACP are similar, and their independent development in different disciplines including the social, natural, and computer sciences reflects the power of the idea and the appeal of transparency in scientific quality assurance.

The review article of Sandewall (2012) outlines and compares further analogies and differences between ETAI and ACP, and it also provides a very useful and comprehensive account of challenges faced by proponents of open peer review. In the following paragraphs I am following up on some of the questions and issues raised.

(1) Defining different types of scientific publication (Sandewall, 2012: p. 2–3): Robust and self-consistent definitions of different types of scientific publications are indeed important for scientific communication and quality assurance. I would, however, not tie such definitions to electronic vs. non-electronic or different types of publishers. Instead, I would suggest to use self-explanatory terms that are meaningful regardless of the publishing medium. Along these lines, the term “discussion paper” has proven well defined and useful as specified in a position statement of the EGU with references to other scientific societies and publishers. Thus, I would recommend broad usage of this term for the first stage of publication in two- or multi-stage open peer review.
(2) Resolving doubts about the viability of open peer review (Sandewall, 2012: Section 4.1): For the reasons outlined by Sandewall (2012) it is important to demonstrate the viability and advantages of open peer review with practical examples. The statistics of ACP and its sister journals prove that the arguments given in Section 4.1 of Sandewall (2012) are valid and applicable to a wide range of research areas involving scientists trained in physics, chemistry, biology, geology, engineering, and other disciplines. Besides a clear concept and terminology (“discussion paper,” etc.), it is important to have a dedicated team of scientists who do not only advertise and explain the new approach but also demonstrate its practical viability by submitting and publishing high quality papers (see below).
(3) Starting the flow of submissions and debate (Sandewall, 2012: Section 4.2): Starting a steady flow of submissions is indeed the most important task for the editorial board of any new journal – even more so for an innovative journal experimenting with new forms of peer review. In most areas of natural science, a journal can be regarded as well established only when it is covered by major indexing services and acquires a journal impact factor or equivalent measure of visibility, which usually takes at least a couple of years. Until then, colleagues without genuine interest in the journal cannot be expected to submit high quality manuscripts that would likely reach higher visibility and citation counts elsewhere. Thus, it is up to the editorial board members and other supporters to maintain a steady flow of high quality submissions. For this purpose as well as for efficient handling of manuscripts when the flow of submissions increases, it is helpful to gather a large editorial board that is firmly rooted in the scientific community and includes experts for all subject areas of the journal scope (ACP: ∼70 board members at the beginning, ∼130 now).
  Initiating the review and discussion of manuscripts with high quality comments that set a precedent for further commenting is of course also important for journals with open peer review. In ACP and its interactive open access sister journals this is mostly done by designated referees appointed by the editor handling the submission. Unsolicited comments can be expected only if members of the scientific community have a strong interest to ask for more information or suggest corrections/additions concerning the methods, results, and conclusions of a study. As expected, non-controversial papers usually receive comments only from the designated referees. Other scientists have little incentive to invest effort and time commenting on papers that they may find potentially useful but not controversial.
(4) Maintaining coherence (Sandewall, 2012: Section 4.3): For ACP, coherence is not more of an issue than for traditional journals covering multiple subject areas with the help of multiple editors. The journal scope has to be well defined and reflect the interests and quality standards of the scientific community served by the journal. Different communities tend to have different standards and preferences with regard to both the format and the content of manuscripts. Therefore, EGU publishes multiple topical journals rather than just one large geosciences journal including all disciplines. Even within the discipline of atmospheric science, EGU publishes more than just one journal, namely ACP and the sister journal “Atmospheric Measurement Techniques” (AMT) which is focused on method development and exhibits similarly high growth rates of volume and visibility as ACP. Due to the transparency of the review process and related self-regulation mechanisms, the quality of final papers published in ACP is generally not more variable than in traditional journals with smaller editorial boards. The ACP editors do not spend extra time on moderating the interactive public discussions, which are not actively moderated for the reasons outlined above. Compared to traditional journals where the editors often rely on simple majority votes of the referees, however, the ACP editors tend to spend more time on carefully validating the referee recommendations, because the transparent review process publicly reveals editorial decisions that are not well-founded.
(5) Computational and administrative infrastructure (Sandewall, 2012: Section 4.4): The installation and maintenance of computational and administrative infrastructure is the main reason why the operation of an open access journal is not cost free, even if most of the review work is done by volunteers. The referees and editors of EGU journals receive no financial rewards. The editors even pay the regular registration fee to participate in the annual EGU General Assemblies with over 10,000 participants where the editorial board meetings take place. The small commercial publisher Copernicus is a spin-off from the Max Planck Society and continues to aim for providing optimal infrastructure and services at minimal cost. Nevertheless, it seems difficult to reduce the average costs far below one thousand Euros per paper, but this is anyhow much lower than the prices of most traditional publishers as discussed above (see Financing and Sustainability of Interactive Open Access Publishing).
(6) Maintaining liveliness of peer review discussion (Sandewall, 2012: see Comparison to Earlier Initiatives with Two- or Multi-Stage Open Peer Review): For the reasons outlined by Sandewall (2012), it is difficult if not impossible to ensure a lively review discussion for all papers published in large scientific journals. This may be problematic for the two-stage review approach of ETAI, where the first stage is designed as a pure community discussion without the involvement of designated referees. For the integrative approach of ACP, however, it is not problematic that most papers receive comments only from the designated referees. The transparency of the peer review process and the option for additional input from the scientific community are sufficient to stimulate self-regulation and enhance the efficiency of scientific quality assurance (Pöschl, 2004, 2010a,b). Discussion papers that report controversial findings often do attract unsolicited comments from the scientific community, but why would researchers invest effort and time in the commenting of their colleagues’ publications which they may find interesting but not controversial? Sometimes more commenting and discussion might be useful, but usually the volume of comments exchanged between authors and referees amounts to as much as 50% of the discussion paper volume, and further commenting can be cumbersome – especially for the authors who normally do not want to spend too much time and effort on the discussion of a single paper but rather move on to the next study. Therefore, unnecessary comments and artificial liveliness of discussion might actually deter authors and do more harm than good to a journal with open peer review.
(7) Open names policy (Sandewall, 2012: Section 7.1): In an ideal world, where people generally react positive to criticism and where scientists can dedicate unlimited amounts of time and effort into compiling completely accurate reviews about their colleagues’ manuscripts, I would agree that referee anonymity should be abandoned. In practice, however, optional anonymity for referees appears appropriate or even necessary to enable critical comments and questions by referees who might be reluctant to risk appearing ignorant or disrespectful (Pöschl, 2004). As outlined above, less than 20% of the referee comments published in the discussion forum of ACP are posted with the name of the referee, i.e., the referees prefer in most cases (>80%) not to reveal their name. Purists often suggest that offering anonymity to referees would be unfair against the authors of a manuscript, and that both parties should be openly named to ensure equal rights and opportunities. They tend to forget, however, that the authors want to get their paper approved by peers, and that the referees usually provide this service on a voluntary basis. In this sense, the authors actually exploit the working capacities of the referees, and the peer review process offers a major gain to the authors (conversion of their manuscript into a peer reviewed paper) but relatively little benefit to the referees. Therefore, it seems appropriate to protect the referees from potential negative consequences of the free service they provided to the authors and to the scientific community. The very small number of author complaints about inappropriate referee comments (about one in 10,000) and the low rejection rates of manuscripts submitted for peer review in ACP and the other EGU interactive open access sister journals (generally <15%) confirm that transparency of the review process (open-process peer review) is normally sufficient to protect authors from inappropriate referee comments. Thus, it seems neither necessary nor appropriate to abandon optional anonymity, impose an open names policy and force referees to reveal their identity. All available evidence suggests that refereeing capacities are the most limited resource in scientific publishing and quality assurance (Pöschl, 2004). In view of the ever-increasing flow of manuscripts submitted for peer reviewed publication, it appears more important to protect referees rather than authors – especially in a multi-stage open peer review process like that of ACP, where the authors anyhow have the opportunity of free speech through their discussion paper and the interactive comments they can post during the open discussion as well as in a final response phase where no more referee comments are allowed^41^.

## Key Questions for Open Evaluation in Science

The coordinators of the special issue hosting this article posed a series of ten key questions to be considered in designing and implementing a concept of open evaluation in science. More than a decade of practical experience and success in re-shaping the processes of scientific publishing and quality assurance as well as continued exchange with scientists and publishing professionals from various disciplines in the sciences and humanities lead to the following answers.

(1) Should some evaluation take place prior to publication or should all evaluation occur post-publication? Experience and rational consideration suggest that the main review process should take place before (final) publication of a manuscript. A fundamental disadvantage of pure post-publication review is that the reviewers cannot contribute to a revision and improvement of the published manuscript. Thus, both the authors and the reviewers are likely to consider critical comments as destructive rather than constructive. Moreover, the reviewer has less incentive to invest effort and time in suggesting additions and corrections, including but not limited to referencing relevant related publications. Last but not least, post-publication commenting does not enhance the information density of scientific communication. If the reviewer comments cannot be implemented in a revised manuscript, the readers have to consult all comments and extract the information from there, which is much less efficient than reading a revised manuscript that synthesizes the information exchanged in the review process. For the above reasons, most publishing platforms that offer only post-publication commenting attract rather small numbers and volumes of comments.
(2) Should reviews and ratings be entirely transparent, or should some aspects be kept secret? Reviews and ratings pertaining to a published manuscript should be made entirely transparent. Reviews and ratings of manuscripts that do not achieve (final) publication, however, should be kept confidential to avoid public escalation of scientific disputes and to give authors a chance of pursuing publication of their (revised) manuscript in alternative publishing venues.
(3) Should alternative metrics, such as paper downloads be included in the evaluation? Paper download statistics are among the many possible forms of post-publication evaluation and should certainly be considered for comprehensive evaluation of scientific publications, but not without precautions against manipulation and misinterpretation of this relatively primitive usage metric. Many scientific journals, including traditional subscription journals with hidden peer review, are already providing download data and highlighting most downloaded papers. This approach certainly facilitates the detection of “hot papers,” but compared to long-term citation statistics and other usage metrics it seems less robust and should not be overrated.
(4) How can scientific objectivity be strengthened and political motivations weakened in the future system? Like in all branches of human society and politics, transparency, and free speech appear to be the best if not the only sustainable way of pursuing objectivity in a balance of powers and interests.
(5) Should the system use signed and authenticated reviews and ratings or anonymous ones, or both? An entirely open and traceable exchange of scientific arguments in the form of signed and authenticated comments is certainly desirable and shall be encouraged. For practical reasons, however, it seems appropriate and beneficial to allow also for anonymous reviews. Optional anonymity enables critical comments and questions by referees who might be reluctant to risk appearing ignorant or disrespectful – especially when providing a voluntary community service in which they have little to gain for investing lots of effort and time.
(6) Should the evaluation be an ongoing process, such that promising papers are more deeply evaluated? The evaluation of scientific publications has to be and generally is an ongoing process – with regard to citation counting as well as commenting and other forms of evaluation that are and have long been in use. Note that also traditional journals with hidden peer review also allow for commentaries referring to earlier papers. In practice, however, relatively few papers seem to attract comments after (final) publication. Moreover, most authors seem to prefer finalizing a publication at some point, and following up with new studies rather than continuously revising and updating old papers. For certain types of publications such as review articles, continuous extension, and revision may be a good and attractive approach as exemplified by the Living Reviews project and journal family^42^. For standard articles presenting new scientific findings, however, a finite process of publication appears more straightforward. Either way, thorough evaluation of scientific studies seems difficult if not impossible without long-term perspective.
(7) How can we bring science and statistics to the evaluation process (e.g., should rating averages come with error bars)? Scientific reviews and ratings are necessarily subject to the same uncertainties and progress as the studies that undergo rating and review. Thus, it seems natural to assess also the reliability of reviews and ratings. One of the many advantages of open peer review is the public availability of reviews and ratings, which makes them accessibly for statistical analysis. Thus open access and open peer review inherently promote the development of new and improved evaluation metrics – in analogy to traditional indexing services like the ISI Web of Science and Elsevier’s SCOPUS, but much more efficiently and comprehensively because of unrestricted access and free competition for optimal solutions.
(8) How should the evaluative information about each paper (e.g., peer ratings) be combined to prioritize the literature? The combination and balancing of different types of evaluative information (ratings/reviews, download/citation statistics, and other usage metrics) will necessarily depend on the aims and perspectives of different types of evaluation or prioritization. For example, the criteria of an evaluation exercise will likely differ for individuals and institutions, scientific researchers and teachers, innovation, and reliability, short-term and long-term impact, etc. In any case, it should be kept in mind statistical indicators are sometimes useful but always also prone to misinterpretation (see publication and citation counting, impact factors, h-indices, etc.).
(9) Should different individuals and organizations be able to define their own evaluation formulae (e.g., weighting ratings according to different criteria)? Obviously, different individuals and institutions may pursue different goals and should thus be able to apply different criteria and weighting schemes. Moreover, evaluators and service providers should compete in developing the best possible metrics and indicators. This is already the case with ISI Web of Science and Elsevier SCOPUS, and through open access and open peer review many more parties can participate, contribute, and help to overcome the obsolete monopoly/oligopoly structures of scientific indexing.
(10) How can we efficiently transition toward the future system? An efficient transition to open evaluation in science can be achieved by combining the strengths of traditional peer review with the opportunities of interactive and transparent community assessment on the internet. The concept of multi-stage open peer review has been designed and successfully applied to induce this transition in the geosciences and is spreading into other disciplines. It can be flexibly adjusted to the needs and peculiarities of different scientific communities, and it has the potential of replacing hidden peer review as the standard of scientific quality assurance and forming the basis of an open evaluation system.

## Conclusions and Outlook

ACP and its sister journals very clearly demonstrate that interactive open access publishing with a multi-stage peer review process effectively resolves the dilemma between rapid scientific exchange and thorough quality assurance. They have proven that multi-stage open peer review indeed fosters scientific discussion, deters submission of sub-standard manuscripts, saves refereeing capacities, and enhances information density in final papers. Moreover, ACP, EGU and Copernicus prove the financial sustainability of open access publishing, and they may serve as a role model for how STM publishing at large can manage the transition from the past of print-based subscription barriers into the future of internet-based open access. The key for a successful, smooth, and efficient transition is to utilize the opportunities of modern technology and interactivity while maintaining the strengths of traditional structures and procedures.

Multi-stage open peer review easily can be integrated into new and existing scientific journals as well as large-scale publishing systems and repositories such as arXiv.org – simply by adding an interactive discussion forum. Equipped with appropriate interactive commenting tools, a large repository such as arXiv.org could not only serve as an archive for “preprints” or “e-prints,” but also as a platform for efficient review and discussion, where authors could post their discussion papers and different journals could send their referees for public review. Similarly, individual publishers could set up central discussion forums to serve different journals or journal sections (Pöschl, 2004, 2010b). This perspective is in line with the selected papers network concept of Lee (2012) and the decoupled journal concept of Priem and Hemminger (2012). Depending on the outcome of public review and discussion, the revised manuscripts could then be sorted and grouped at different levels of relevance for different audiences – analogous to the quality ranking system and tiers of the Berkeley Electronic Press journals in economics^43^^,^^44^. Another feature that could be integrated in multi-stage open peer review is a double-blind approach in the initial access review (pre-screening) to avoid/minimize bias in selection of discussion papers. In the open discussion, however, it seems more useful and efficient to discuss openly without hiding identities (except for protecting referees if they wish to stay anonymous).

For interdisciplinary highlight papers, EGU and Copernicus are currently preparing the introduction of a third stage of interactive open access publishing that shall lead to efficient grouping of scientific publications in three tiers with the following characteristics:

1. Discussion forum (discussion papers and interactive comments):
  – free speech (for authors and scientific community)
  – original opinions
  – immediate publication and dissemination
2. Topical journal (final papers):
  – thorough quality assurance (collaborative peer review)
  – comprehensive, complete and validated information
3. Highlight magazine (abstracts):
  – highly condensed information
  – interdisciplinary relevance and public interest
  – three-stage selection process (distillation).

The interactive open access highlight magazine shall be dedicated to the selection and presentation of the abstracts of highlight papers, which outline the forefront of research and are of high interdisciplinary relevance and public interest. The editorial board of the magazine shall select highlight papers that have undergone public peer review and discussion in topical open access journals, and the abstracts of the highlight papers shall be commented and compiled with direct references and links to the original papers and journals, respectively. By building on rather than competing with topical scientific journals, the highlight selection process and magazine shall provide high efficiency, conciseness and interdisciplinarity without compromising scientific completeness and quality assurance. This might also be a way forward for traditional highlight magazines like Nature or Science covering the full width of scientific disciplines.

The basic concepts of interactive open access publishing and peer review can be easily adjusted to the different needs and capacities of different scientific communities by maintaining or abandoning referee anonymity, shortening, or prolonging the public discussion phase, adding post-peer review commenting and rating tools for readers, making all steps/iterations of peer-review and revision transparent, adding further stages of publication for re-revised manuscripts, establishing feedback loops for editorial quality assurance, etc.

**Figure 3:**
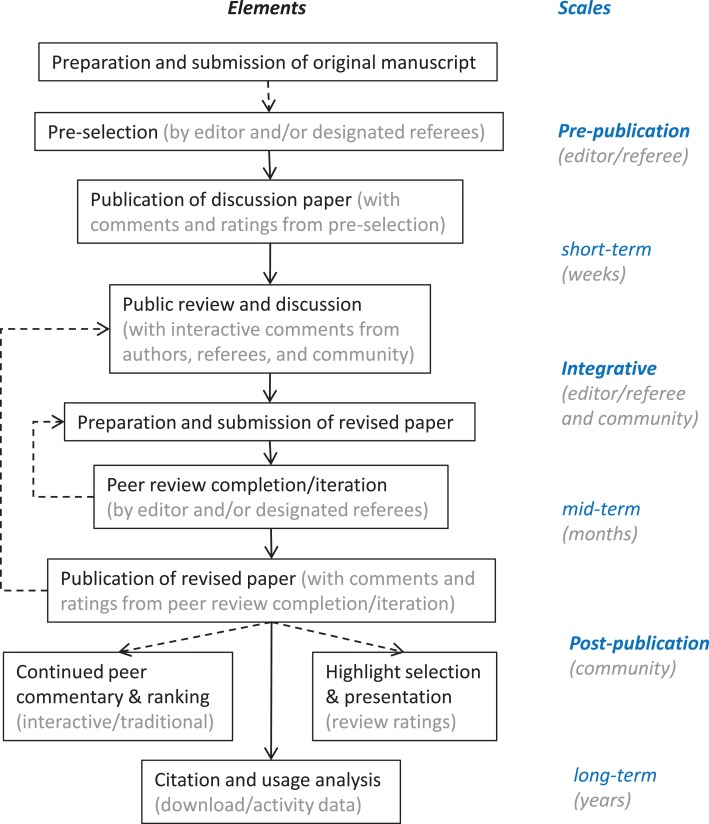
**Elements and scales of evaluation in an open system of scientific publication and quality assurance based on multi-stage open peer review**. Solid and dashed arrows indicate essential and optional processes.

Figure 3 illustrates essential elements and scales of evaluation in an open system of scientific publication and quality assurance based on multi-stage open peer review. While much of the general discussion about reforming scientific quality assurance and evaluation is focused on a distinction of pre- and post-publication processes, the experience and achievements of ACP and EGU show that an integrative approach combining pre- and post-publication elements in a multi-step process of review and publication is most efficient.

Besides communication and evaluation of scientific results, multi-stage open peer review might also be applicable for efficient evaluation of scientific research proposals in the form of citable discussion papers. Again all involved parties could profit from public documentation, scrutiny, and citability. At first sight, it might appear that the authors of a proposal would run a high risk of “losing” innovative project ideas to the public. In practice, however, they might be better protected from (hidden) plagiarism and obstruction by competitors, and the citable publication might actually help them to claim authorship, precedence, and recognition for their ideas. At the same time, the scientific community and society at large might profit from rapid dissemination of innovative ideas.

Overall, interactive open access publishing and peer review can strongly enhance scientific exchange and quality assurance. The concept has been very successfully applied and extended over the past decade, demonstrating both the scientific benefits and the financial sustainability of open access. It will likely emerge as a best practice model for the future of scientific publishing, and it provides a solid basis for efficient use and augmentation of scientific knowledge in the global information commons (David and Uhlir, 2005). Moreover, public review, discussion, and documentation of the scientific discourse can serve as an example for rational and transparent procedures of settling complex questions, problems, and disputes. It is a model for further development of the structures, mechanisms, and processes of communication and decision making in society and politics in line with the principles of critical rationalism and open societies.

A major limiting factor for the development of innovative scientific publication and evaluation systems is the scarcity of funds specifically dedicated to covering open access publication costs. Nevertheless, more and more funding agencies do provide funds for this purpose, and the success of the EGU/Copernicus as well as other open access publishers shows that many scientists are willing and able to cover the costs of open access publishing via publication fees. Overall, the money required to produce scientific publications in a format that is accessible via the internet is already in circulation. Otherwise, the publishers would not be able to offer online subscriptions. Currently, however, the funds are channeled through a rigid subscription system, which has the consequence that certain publishers can make excessively large profits and that the scientific information remains locked away. If the same amount of money were channeled through a flexible open access funding schemed, the same products (scientific journals and papers) could be produced and made freely available on the internet at the same or lower cost in a proper publishing market rather than the current subscription scheme with oligopoly character.

In order to accelerate the improvement of scientific communication and evaluation in a global information commons, I would like to renew the following propositions and recommendations to scientists and scientific publishers, librarians, institutions, and funding agencies (Pöschl, 2004, 2010b):

1. Promote open access to publicly funded research publications by appropriate guidelines and by moving funds from subscription budgets to publication budgets – preferably at high rates (20% per year or more). Obviously, traditional publishers are reluctant to undermine their profits as long as they can rely on rigid subscription schemes, but the ones who are ready to serve science will swiftly adapt to new financing schemes as illustrated by the open choice model and acquisition of BioMedCentral by Springer^45^. The others can be substituted by new service providers as indicated by the swiftly growing number, size, and visibility of open access publishers and journals^46^^,^^47^.
2. Promote multi-stage open peer review in new and existing journals, repositories, and other publication platforms. Public review and interactive discussion are technically straightforward and can be flexibly adjusted to different scientific communities, but care should be taken when dealing with key features of peer review and scientific discourse (optional anonymity for designated referees, permanent archiving, and citability of published manuscripts and comments, etc.).
3. Promote the development and implementation of new and improved metrics for the impact and quality of scientific publications (combination of citation, download/usage, commenting, and ranking by various groups of readers and users, respectively). Note that open access is urgently needed to stimulate innovation by competition in this field, which has long been hampered by monopoly structures. The working capacities of librarians and related information professionals that may be liberated by the end of the subscription business are urgently needed for the structuring, processing, quality assurance, and digital preservation of scientific contents, bibliometric data, and statistical analyses both at scientific institutions and at commercial service providers.

## Conflict of Interest Statement

The author declares that the research was conducted in the absence of any commercial or financial relationships that could be construed as a potential conflict of interest.

## Footnotes

^1^www.atmos-chem-phys.net

^2^www.egu.eu

^3^www.copernicus.org

^4^http://www.atmospheric-chemistry-and-physics.net/general_information/public_relations.html

^5^http://www.atmos-chem-phys-discuss.net/most_commented_papers.html

^6^www.publications.copernicus.org/open_access_journals/journals_by_subject.html

^7^www.atmospheric-chemistry-and-physics.net

^8^www.atmospheric-measurement-techniques.net

^9^www.biogeosciences.net

^10^www.climate-of-the-past.net

^11^www.drinking-water-engineering-and-science.net

^12^www.earth-system-dynamics.net

^13^www.earth-system-science-data.net

^14^www.geoscientific-model-development.net

^15^www.hydrology-and-earth-system-sciences.net

^16^www.ocean-science.net

^17^www.social-geography.net

^18^www.solid-earth.net

^19^www.the-cryosphere.net

^20^http://www.egu.eu/publications/open-access-journals.html

^21^www.economics-ejournal.org

^22^www.agu.org, since 2008

^23^www.plosone.org, since 2007

^24^www.biology-direct.com, since 2006

^25^http://www.etaij.org/, since 1997

^26^http://www-jime.open.ac.uk, since 1996

^27^www.mpg.de

^28^www.insu.cnrs.fr/

^29^www.biomedcentral.com

^30^www.bbsonline.org

^31^psycprints.ecs.soton.ac.uk

^32^www.nature.com/nature/peerreview/debate/nature05535.html

^33^http://precedings.nature.com/site/help

^34^http://www.atmospheric-chemistry-and-physics.net/general_information/faq.html

^35^http://www.egu.eu/statements/position-statement-on-the-status-of-discussion-papers-published-in-egu-interactive-open-access-journals-4-july-2010.html

^36^www.biology-direct.com/info/about/

^37^www.atmospheric-chemistry-and-physics.net/review/ms_evaluation_criteria.html

^38^www.atmos-chem-phys-discuss.net/8/S12406/2009

^39^www.biomedcentral.com

^40^www.insu.cnrs.fr/

^41^http://www.atmospheric-chemistry-and-physics.net/review/review_process_and_interactive_public_discussion.html

^42^http://www.livingreviews.org/

^43^www.bepress.com/bejm

^44^www.bepress.com/bejte

^45^http://www.springer.com/open + access

^46^www.oaspa.org

^47^www.doaj.org
